# Principles, Applications, and Biosafety of Plant Genome Editing Using CRISPR-Cas9

**DOI:** 10.3389/fpls.2020.00056

**Published:** 2020-02-13

**Authors:** Kaoutar El-Mounadi, María Luisa Morales-Floriano, Hernan Garcia-Ruiz

**Affiliations:** ^1^ Department of Biology, Kuztown University of Pennsylvania, Kuztown, PA, United States; ^2^ Recursos Genéticos y Productividad-Genética, Colegio de Postgraduados, Texcoco, Montecillo, Mexico; ^3^ Department of Plant Pathology and Nebraska Center for Virology, University of Nebraska-Lincoln, Lincoln, NE, United States

**Keywords:** genome editing, CRISPR-Cas9, Cas9 human exposure, plant breeding, biosafety regulations

## Abstract

The terms genome engineering, genome editing, and gene editing, refer to modifications (insertions, deletions, substitutions) in the genome of a living organism. The most widely used approach to genome editing nowadays is based on Clustered Regularly Interspaced Short Palindromic Repeats and associated protein 9 (CRISPR-Cas9). In prokaryotes, CRISPR-Cas9 is an adaptive immune system that naturally protects cells from DNA virus infections. CRISPR-Cas9 has been modified to create a versatile genome editing technology that has a wide diversity of applications in medicine, agriculture, and basic studies of gene functions. CRISPR-Cas9 has been used in a growing number of monocot and dicot plant species to enhance yield, quality, and nutritional value, to introduce or enhance tolerance to biotic and abiotic stresses, among other applications. Although biosafety concerns remain, genome editing is a promising technology with potential to contribute to food production for the benefit of the growing human population. Here, we review the principles, current advances and applications of CRISPR-Cas9-based gene editing in crop improvement. We also address biosafety concerns and show that humans have been exposed to Cas9 protein homologues long before the use of CRISPR-Cas9 in genome editing.

## Introduction

The world population is predicted to reach 10 billion by 2050. While the available farm land and water are being reduced, the global demand for food will increase 25%–70% above current production levels (Hunter et al., 2017). Thus, feeding a rapidly growing population, particularly in the face of climate change, is a big challenge. There is, therefore, an urgent need to improve food production and accelerate sustainable agricultural development.

Long time before the field of genetics was established, humans genetically modified plants through breeding and selection. Without knowledge of genes, mutagenesis, or gene editing, our ancestors influenced the genetic make-up of plants and animals by selecting for traits conducive to food production (Wang et al., 1999; Clark et al., 2005; Li et al., 2013b). A prime example is maize (*Zea mays subsp. mays L.*), which is one of the most produced cereals worldwide. Molecular, cytological, and isozyme profiles have shown that maize is a descendant of an annual species of teosinte (*Zea mays* ssp. *parviglumis*) native to the Balsas River Valley on the Pacific slopes of the states of Michoacán and Guerrero, Mexico. The process started approximately 9,000 years ago. Teosinte has a popping ability that provided an incentive for its cultivation. Repetitive cycles of selection for traits conducive to kernel production led to the development of the maize plant as we know it (Doebley et al., 1990; Dorweiler et al., 1993; Piperno and Flannery, 2001).

To make plant breeding faster, more predictable, and amendable to a wide range of species, several techniques of plant genetic engineering have been developed. Genome editing through programmable endonucleases is the most recent approach to genetic engineering. Endonucleases are used to specifically induce double strand breaks in target genes of interest. The cellular DNA repair pathway then acts on the double strand break to restore the damage through non-homologous end joining (NHEJ) or homology-directed repair (HDR). In the process, insertions, deletions, substitutions, and DNA recombination may occur (Puchta et al., 1996; Puchta, 2005; Symington and Gautier, 2011).

Three kinds of programmable endonucleases are currently being used for plant genome editing. Zinc finger nucleases, transcription activator-like effector nucleases (TALENs), and CRISPR-Cas9 (Malzahn et al., 2017; Shah et al., 2018; Zhang et al., 2018a; Bao et al., 2019). Zinc finger nucleases are chimeric proteins composed of a synthetic zinc finger DNA binding domain and a DNA cleavage domain. The zinc finger DNA binding domain can be modified to specifically target any long stretch of double stranded DNA of interest (Kim et al., 1996; Cathomen and Joung, 2008). Zinc finger nucleases have been used to edit the genomes of several species, including maize, rice and Arabidopsis (Shukla et al., 2009; Osakabe et al., 2010; Ainley et al., 2013; Gallego-Bartolome et al., 2019).

TALENs are sequence-specific nucleases consisting of transcription activator-like effectors fused to the catalytic domain of the *FokI* endonuclease (Boch et al., 2009; Christian et al., 2010). The DNA-binding domain in TALE monomers in turn is comprised of a central repeat domain (CRD) that directs DNA binding and host specificity. The CRD is formed by tandem repeats of 34 amino acid residues, each binding to one nucleotide in the target nucleotide sequence which allows more flexible target design and increases the number of potential target sites relative to those that can be targeted by zinc finger nucleases (Moscou and Bogdanove, 2009). Genome editing by TALENs has been demonstrated in a wide variety of plants including Arabidopsis (Christian et al., 2013), barley (Budhagatapalli et al., 2015), Brachypodium (Shan et al., 2013), maize (Char et al., 2015), tobacco (Zhang et al., 2013), potato (Clasen et al., 2016; Nicolia et al., 2015), rice (Li et al., 2012; Shan et al., 2013; Shan et al., 2015), soybean (Du et al., 2016), sugarcane (Jung and Altpeter, 2016), tomato (Lor et al., 2014), and wheat (Liang et al., 2014).

**Figure 1:**
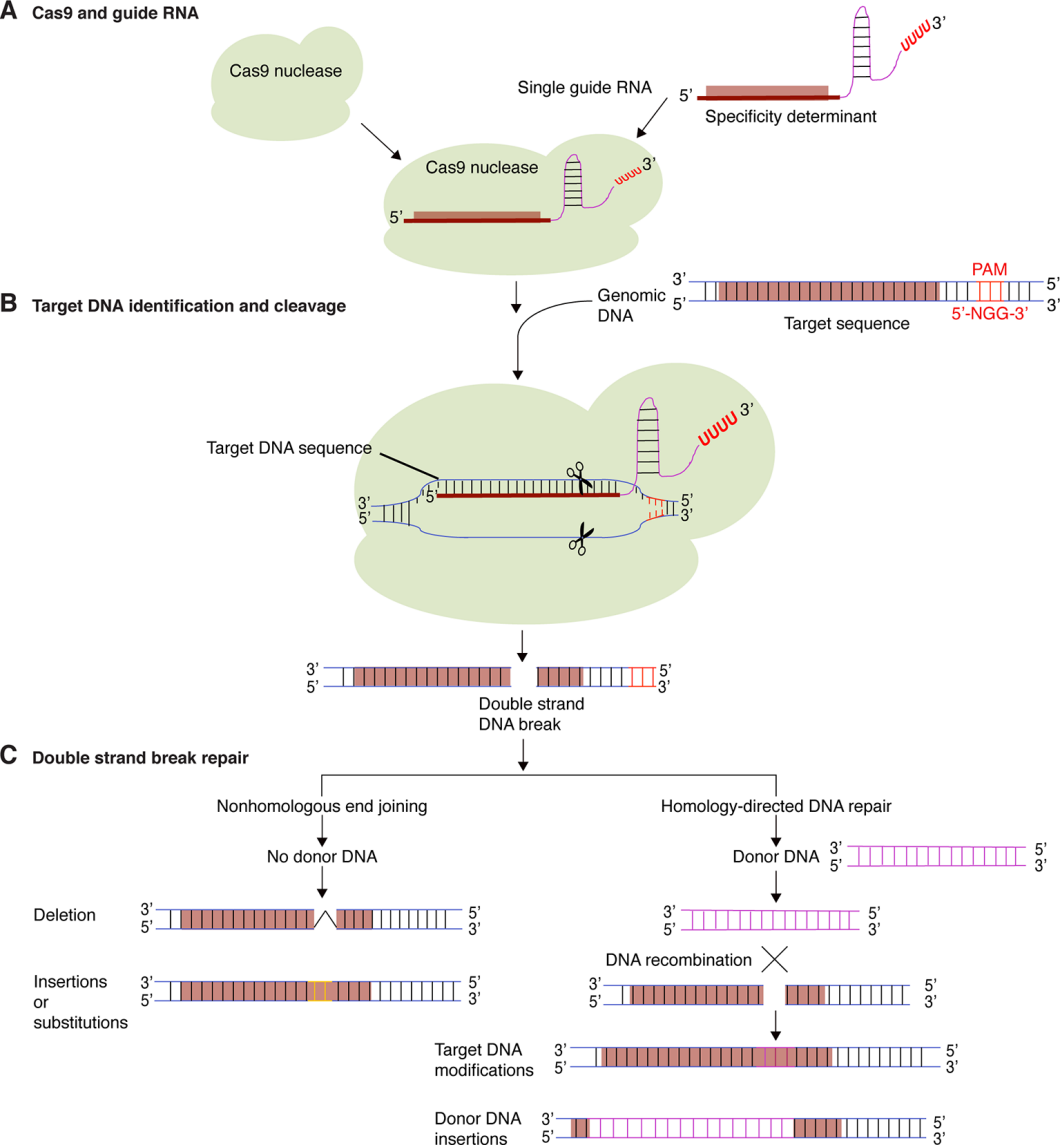
Targeted genome editing using CRISPR-Cas9. **(A)** The CRISPR-Cas9 system consists of a Cas9 protein and one or several guide RNA. Guide RNAs determine target DNA specificity by sequence complementarity**. (B)** Guide RNA and Cas9 protein form a binary complex that specifically cleaves target DNA creating a double-strand DNA break. **(C)** Cellular DNA repair mechanisms, non-homologous end joining (NHEJ) and homology-directed repair (HDR), repairs the double-strand DNA break. In the process, short insertions, deletions, nucleotide substitutions, or gene insertion may occur.

The CRISPR-Cas9 system consists of a programmable Cas9 nuclease and a synthetic short guide RNA (sgRNA). DNA target specificity is provided by the guide RNA (**Figure 1**). Thus, the CRISPR-Cas9 system is much easier to be constructed than Zinc finger or TALENs, simple, efficient, has low cost and allows the targeting of multiple genes at once (Cong et al., 2013; Mali et al., 2013).

The CRISPR-Cas9 system has a wide diversity of applications. In medicine, it has been applied in research related to cancer, virus infections, genetic diseases and detection of pathogens. This system has been successfully used in mice to correct mutations in monogenic diseases (Schwank et al., 2013; Ye et al., 2014; Yin et al., 2014; Kang et al., 2015), including the one responsible for Duchenne muscular dystrophy (DMD) (Long et al., 2016; Nelson et al., 2016; Tabebordbar et al., 2016). CRISPR-Cas9 has also been used to disrupt HIV-1 provirus (Ebina et al., 2013), human papillomaviruses (Kennedy et al., 2014) and hepatitis B virus (Kennedy et al., 2015). Furthermore, CRISPR-Cas9 has also been used to target human hereditary liver diseases (Yang et al., 2016; Yin et al., 2016) and has shown great promise for the treatment of cancer (Chen et al., 2019) and Hutchinson–Gilford progeria syndrome (Beyret et al., 2019). In human cells, CRISPR-Cas9 has been used successfully to replace endogenously-encoded antibodies with antibodies protective against Respiratory Syncytial Virus (RSV), human immunodeficiency virus (HIV), influenza virus, and Epstein-Barr virus (EBV) (Moffett et al., 2019). This review is focused on applications of CRISPR-Cas9 in crop improvement.

## Components of CRISPR-CAS9

Clustered regularly interspaced short palindromic repeats (CRISPR) are a family of DNA sequences found in the genomes of bacteria and archaea. CRISPRs were first discovered downstream of the alkaline phosphatase isozyme gene (*iap)* in *Escherichia coli* (Ishino et al., 1987). Palindromic repeats are separated by short (32 to 36 bp) sequences derived from the DNA of viruses that have previously infected the cell or its predecessors. These virus-derived sequences integrated into the bacterial genome provide a memory system of previous virus infection. Once integrated into the genome, CRISPRs are transcribed and the virus-derived sequences form short guide RNAs that are bound by CRISPR associated protein 9 (Cas9). Cas9 is a DNA endonuclease. In bacteria and archaea, the natural role of the CRISPR-Cas9 system is to provide adaptive antiviral immunity against DNA viruses. Binary complexes formed by guide RNA-Cas9 recognize and cleave DNA of incoming viruses with sequence similarity to the guide RNA (Garneau et al., 2010; Horvath and Barrangou, 2010; Jinek et al., 2012; Sternberg et al., 2014).

There are several CRISPR-Cas systems in bacteria and archaea. For a comprehensive review, see (Karginov and Hannon, 2010; Sorek et al., 2013). The CRISPR-Cas9 system most frequently used in plant genome editing is an adaptation of the type II CRISPR-Cas system of *Streptococcus pyogenes* (Garneau et al., 2010). *S. pyogenes* is a Gram-positive human-restricted pathogen that colonizes the pharynx and the skin causing an array of diseases ranging from mild sore throat and impetigo to invasive and life-threatening infections (Cunningham, 2000; Rosinski-Chupin et al., 2019). Type II CRISPR-Cas system consists of a Cas9 protein with DNA endonuclease activity and one CRISPR RNA transcript that is processed to form one or several short guide RNAs that direct Cas9 to the target DNA sequence (**Figure 1**) (Jinek et al., 2012; Lander, 2016; Jiang and Doudna, 2017). In the cell, Cas9 binds to the guide RNA and forms a binary complex that scans the genome for the DNA target for cleavage using Watson-Crick base pairing. The specificity is determined by the guide RNA. Cas9 also requires a specific proto-spacer adjacent motif (PAM) localized on the non-target DNA strand, directly downstream of the target DNA sequence (**Figure 1B**). Cas9 from *S. pyogenes* recognizes NGG as a PAM (Anders et al., 2014; Lander, 2016; Jiang and Doudna, 2017). Cas9 proteins have two signature nuclease domains: HNH and RuvC. The HNH-like nuclease domain cleaves the target DNA strand complementary to the guide RNA sequence. The RuvC-like nuclease domain cleaves the non-target strand (Jinek et al., 2012; Gao et al., 2017; Jiang and Doudna, 2017). This creates a DNA double strand break (DSB) at the target site, which can be subsequently used to introduce modifications by NHEJ or HDR (**Figure 1C**) (Symington and Gautier, 2011). In higher plants, NHEJ occurs most frequently than the more precise HDR, which requires a donor DNA template during homologous recombination to repair the dsDNA breaks. NHEJ does not require a homologous repair template (Puchta et al., 1996; Puchta, 2005). NHEJ has therefore become a popular way to disrupt genes by the creation of small base pair indels (insertions/deletions) at specific points in the target genes, while HDR can be used to precisely introduce specific point mutations and insert or replace desired sequences into the target DNA (**Figure 1C**) (Li et al., 2013a). Currently, it is also possible to precisely edit a single base pair in the genome without the introduction of DSBs by using engineered Cas9 base editors. The base editors consist of a dead Cas9 domain fused to a cytidine deaminase enzyme that can be programmed with a guide RNA and is able to convert G to A and C to T without inducing dsDNA breaks (Komor et al., 2016). A Cas9 fused with a transfer RNA adenosine deaminase that can mediate conversion of G to A and C to T was also created (Gaudelli et al., 2017). These base editors install point mutations without generating excess undesired editing byproducts. In plants, base editing has been used to efficiently generate point mutations in maize, rice and wheat (Rees and Liu, 2018). These base editors will allow more and better genome modifications and expand the type of cells that can be efficiently edited. In order to make CRISPR-Cas9 a successful genome editing technology in plants, several modifications have been implemented. These include codon modification of the protein Cas9 to ensure its stability in plants, the use of strong constitutive or inducible promotors and the development of versatile DNA cassettes to co-express guide RNAs and Cas9 in the same cells (Li et al., 2013a).

## The Genome Editing Process

A fundamental part of the genome editing process is the identification of target genes that determine phenotypes of interest, such as susceptibility to viruses (Garcia-Ruiz, 2018), other pathogens, resistance to herbicides or adverse environmental factors (**Table 1**). Assessment of natural variation or systematic genome-wide screens are also powerful approaches to identify target genes (Kushner et al., 2003; Panavas et al., 2005; Pyott et al., 2016; Giner et al., 2017).

**Table 1:** Representative applications of CRISPR-Cas9 in crop breeding.

Group	Crop species	Target gene	Role	Modification	Target trait	Reference
Monocotyledon	Maize	*ZmTMS5*	Causes the TGMS trait	Gene knockout	Thermosensitive genic male sterility	(Li et al., 2017)
Monocotyledon	Sorghum	*k1C*	Encode 22-kD α-kafirin proteins	Genes disruption in N-terminal ER signal peptide region	High Lysine content and increased protein digestibility	(Li et al., 2018a)
Monocotyledon	Wheat	*TaEDR1*	Negative regulator of the defense response against powdery mildew	Knock-down all three homologs of *TaEDR1*	Powdery mildew resistance	(Zhang et al., 2017b)
Monocotyledon	Wheat	*TaGW2-A1, -B1 and -D1.*	Genetic control of grain weight and protein content traits	Homologous genes knockout	Grain weight and protein content increase	(Zhang et al., 2018a)
Monocotyledon	Wheat	*Ms1*	Male fertility gene	Gene knockout	Male sterility	(Okada et al., 2019)
Monocotyledon	Rice	*OsRR22*	Transcription factor	Inactivating mutations	Enhanced salinity tolerance	(Zhang et al., 2019a)
Monocotyledon	Rice	*CAO1 and LAZY1*	Synthesis of Chl b from Chl a and regulating shoot gravitropism, respectively	Genes' disruption	Defective synthesis of Chlorophyll b and tiller-spreading phenotypes	(Miao et al., 2013)
Monocotyledon	Rice	*SBEI and SBEIIb*	Determining the amylose content, fine structure of amylopectin, and physiochemical properties of starch	Genes disruption	Higher proportion of long chains in amylopectin	(Sun et al., 2017)
Monocotyledon	Rice	*Gn1a, DEP1, GS3 and* IPA1	Regulators of grain number, panicle architecture, grain size and plant architecture, respectively	Genes disruption	Enhanced grain number, dense erect panicles, and larger grain size, respectively	(Li et al., 2016)
Monocotyledon	Rice	*OsERF922*	Negative regulator of Rice blast resistance	Gene disruption	Enhanced rice blast resistance	(Wang et al., 2016a)
Monocotyledon	Rice	*OsSWEET13*	Sucrose transporter. Negative regulator of bacterial blight resistance	Gene knockout	Bacterial blight resistance	(Zhou et al., 2015)
Monocotyledon	Rice	*OsMATL*	Encodes a pollen-specific phospholipase	Gene knockout	Haploid seed formation	(Yao et al., 2018)
Monocotyledon	Rice	*ALS*	Acetolactate synthase encoding gene	Gene disruption	Herbicide resistance	(Endo et al., 2016)
Monocotyledon	Rice	*ALS*	Acetolactate synthase encoding gene	Gene replacement	Herbicide resistance	(Sun et al., 2016)
Monocotyledon	Rice	*TMS5*	Thermo-sensitive genic male sterility gene	Gene knockout	Thermo-sensitive genic male sterility	(Zhou et al., 2016)
Monocotyledon	Cavendish banana *Musa acuminata*)	*PDS*	Phytoene desaturase encoding gene	Gene knockout	Albinism phenotype	(Naim et al., 2018)
Monocotyledon	Banana *(Musa spp.)*	Integrated endogenous *banana streak virus* (eBSV) in the B genome of plantain	The eBSV activates into infectious viral particles under stress	Knockout the integrated dsDNA of BSV from the banana genome	Asymptomatic plants to *banana streak virus*	(Tripathi et al., 2019)
Dicotyledon	*Camelina sativa*	*FAD2*	Fatty acids biosynthesis	Genes knockout	Improve seed Oleic acid content	(Jiang et al., 2017)
Dicotyledon	*Arabidopsis thaliana*	The *FWA* and the *SUPERMAN* promoters.	Flowering time gene and a transcriptional regulator of floral homeotic genes	Genes knock in.	Targeted gene activation and DNA methylation in Arabidopsis	(Papikian et al., 2019)
Dicotyledon	*Arabidopsis thaliana*	*CBFs*	C-repeat binding factors encofing genes, key transcription factors in the cold stress response	Genes disruption. Deletions and insertions	Cold tolerance	(Jia et al., 2016b)
Dicotyledon	Tomato	*SlJAZ2*	Important repressor in jasmonate signaling pathway. Key regulator of stomatal aperture during biotic stresses	Gene knock in, lacking the C‐terminal Jas domain	Bacterial speck resistance	(Ortigosa et al., 2018)
Dicotyledon	Tomato	*SlMlo1*	Confers susceptibility to fungi, causing the powdery mildew disease	Gene disruption. 48 bop deletion	Powdery mildew resistance	(Nekrasov et al., 2017)
Dicotyledon	Tomato	*SP5G*	Florigen paralog and flowering repressor	Gene knockout	Rapid flowering. Early yield	(Soyk et al., 2017)
Dicotyledon	Tomato	*SlAGL6*	Transcription factor. It plays essentials roles, especially in flower meristem and floral organ development	Gene knockout	Parthenocarpic phenotype	(Klap et al., 2017)
Dicotyledon	Tomato	*SlIAA9*	Key gene controlling parthenocarpy	Gene knockout	Parthenocarpic phenotype	(Ueta et al., 2017)
Dicotyledon	Tomato	*SlMAPK3*	Mitogen-activated protein kinases 3 encoding gene, responds to drought stress	Gene knockout.	Drought tolerance	(Wang et al., 2017)
Dicotyledon	Tomato	*CrtR*-*b2* and *Psy1.*	Key genes of carotenoid biosynthesis	Genes knockout	Changes on carotenoids profile	(D'ambrosio et al., 2018)
Dicotyledon	Wild tomato	*SELF-PRUNING, OVATE, FASCIATED and FRUIT WEIGHT 2.2, MULTIFLORA and LYCOPENE BETA CYCLASE*	Encode general plant growth habit, fruit shape, fruit size, fruit number and nutritional quality, respectively	Genes knockout	Obtain domestication traits (fruit number, size, shape, nutrient content and plant architecture)	(Zsogon et al., 2018)
Dicotyledon	Stress-tolerant wild-tomato	*SP, SP5G, SlCLV3, SlWUS and SlGGP1*	Flowering repressors, small-peptide-encoding gene, homeobox-encoding gene and vitamin C–biosynthetic enzyme encoding gene.	Genes disruption. Insertions, deletions and invertions.	Domesticated phenotypes yet retained parental disease resistance and salt tolerance	(Li et al., 2018b)
Dicotyledon	Potato	*GBSS*	Granule-bound starch synthase encoding gene, is responsible for amylose synthesis	Gene knockouts	Increased amylopectin content	(Andersson et al., 2017)
Dicotyledon	Cucumber	*eIF4E*	Eukaryotic translation initiation factor. Is a central part of the translation machinery	Gene knockout	Cucumber Vein Yellowing Virus, Zucchini yellow mosaic virus and Papaya ring spot mosaic virus-W resistance	(Chandrasekaran et al., 2016)
Dicotyledon	Soybean	*GmFT2a*	Integrator in the photoperiod flowering pathway in soya bean	Gene disruption.1‐bp insertion or short deletion	Late flowering	(Cai et al., 2018)
Dicotyledon	Grape	VvWRKY52	Transcription factor gene that plays important roles in plant defense regulatory networks in grape	Gene knockout	*Botrytis cinerea* resistance	(Wang et al., 2018)
Dicotyledon	Oranges	*CsLOB1*	Plays a critical role in promoting pathogen growth and erumpent pustule formation	Disruption of *CsLOB1* promoter. Deletions, insertions and substitutions	Citrus canker resistance	(Peng et al., 2017)
Dicotyledon	Grapefruits	*CsLOB1*	Critical citrus disease susceptibility gene for citrus canker	Disruption the coding region of both alleles of *CsLOB1*	Citrus canker resistance	(Jia et al., 2017)
Dicotyledon	Grapefruits	*CsLOB1*	Plant‐specific transcriptional factor in the lateral organ boundaries (LOB) domain family	Disruption of the PthA4 effector binding elements in the Type I CsLOB1 Promoter	Citrus canker alleviated	(Jia et al., 2016a)
Fungus	Mushroom	*PPO*	Enzymes that use molecular oxygen to oxidize *ortho*-diphenols to *ortho*-quinones. These commonly cause browning reactions following tissue damage, and may be important in plant defense. Some PPOs function as hydroxylases	Knockout of one of six *PPO* genes	Non-browning phenotype	(Waltz, 2016b)

**Figure 2:**
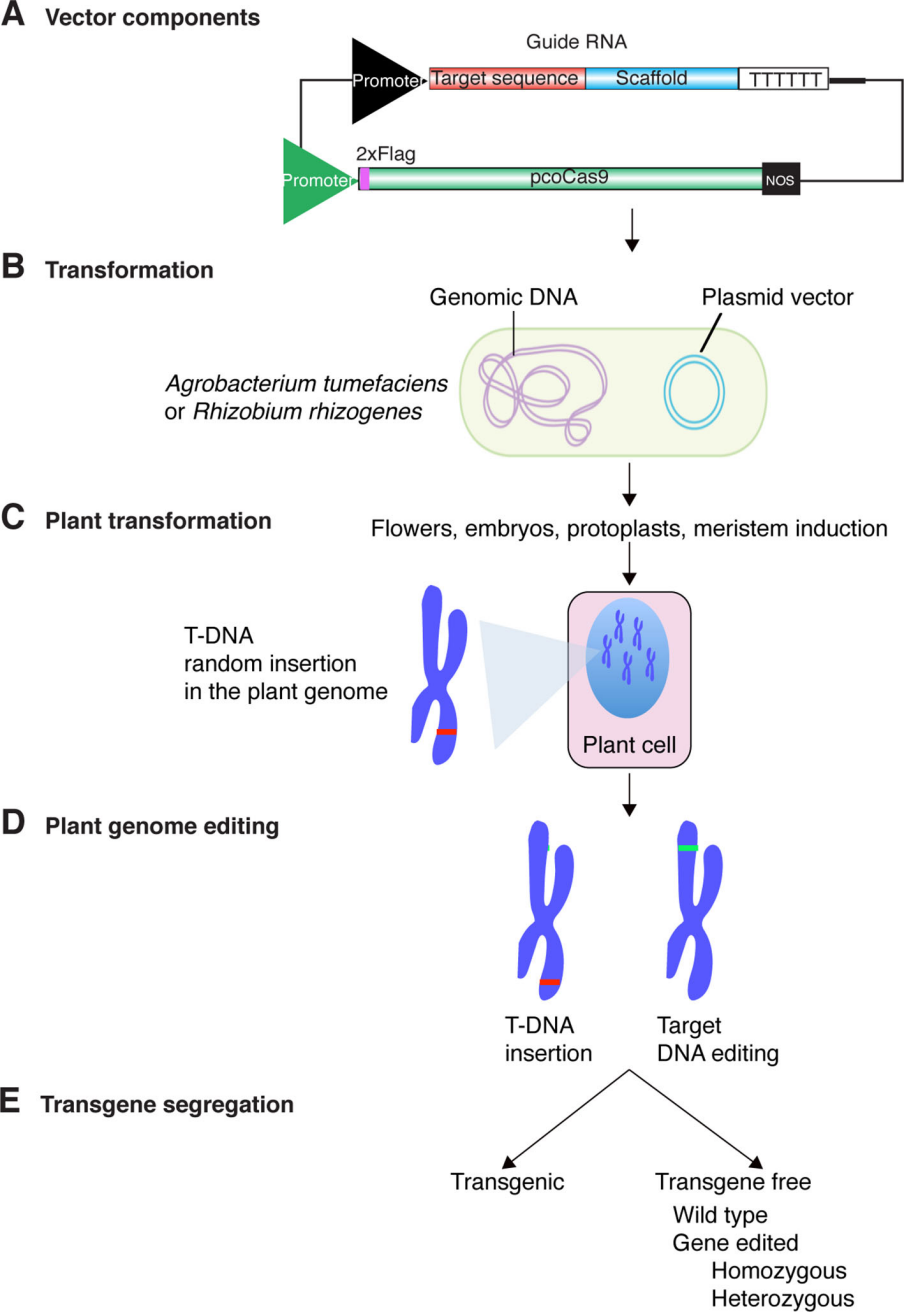
Genome editing process using CRISPR-Cas9 and Agrobacterium tumefaciens. **(A)** Cas9 protein and guide RNAs are cloned into the same plasmid vector containing transfer DNA (T-DNA) signals. Expression is driven by strong constitutive (U6, 35S, or other), inducible or tissue specific promoters. Transcription termination is programmed by addition of terminator such as the U6 or Nopaline synthase (NOS). For plant genome editing purposes, Cas9 has been codon-optimized and might contain an epitope tag to determine expression. **(B)** *A. tumefaciens* or *R. rhizogens* is transformed with the plasmid vector carrying the cassette for Cas9 protein and guide RNAs expression. **(C)** Bacteria is used to transform embryos, ovules in flowers, protoplasts, roots, or cells in leaves. Integration site of the T-DNA is random. **(D)** Expression of Cas9 protein and guide RNAs lead to editing of the target DNA. The T-DNA insertion site and the DNA target are likely not linked. **(E)** The T-DNA insertion and edited part of the genome can be separated by Mendelian segregation.

Guide RNAs are artificially designed to specifically direct Cas9 to the target gene to be edited. Bioinformatic programs that generate candidate guide RNAs while accounting for the possibility of off-targets are available (http://crispr.mit.edu/). Dynamic expression vectors have also been designed to clone and co-express guide RNAs and Cas9 (Li et al., 2013a; Fauser et al., 2014; Shimatani et al., 2017). Although variations have been developed recently (Toda et al., 2019), transformation of plant cells to express guide RNAs and Cas9 follows a process similar to these established for the generation of transgenic plants (**Figure 2**). The expression cassettes contain constitutive or inducible promoters, transcription terminators and antibiotic and/or herbicide resistance markers used for selection purposes (Li et al., 2013a; Fauser et al., 2014; Shimatani et al., 2017).

The vector carrying the Cas9 protein and the guide RNA is then introduced into *Agrobacterium tumefaciens* or *Rhizobium rhizogenes* (**Figure 2B**). Colonies containing the CRISPR-Cas9 construct are further used to transform plants by *Agrobacterium*-mediated transformation and first generation transgenic plants are identified by antibiotic or herbicide selection (Li et al., 2013a; Pyott et al., 2016; Veillet et al., 2019). Green fluorescent protein (GFP) has also been used to distinguish cells or calluses containing the CRISPR-Cas9 cassette (Doench et al., 2014). In all cases, sequencing the target gene is required in order to identify the mutations introduced by genome editing. The presence of the CRISPR-Cas9 cassette renders the plants transgenic and thus subject to the corresponding biosafety regulations (Callaway, 2018; Garcia Ruiz et al., 2018; Eckerstorfer et al., 2019). However, in sexually propagated plants, after identification of the genome edited plants, the CRISPR-Cas9 transgene can be eliminated by Mendelian segregation (**Figures 2D–E**) (Zhang et al., 2019a). This key part of the process removes the transgene in the third or subsequent generations resulting in the formation of genome-edited plants without a transgene (Pyott et al., 2016). Because of the absence of the transgene in these plants, they resemble those with mutations generated by natural means or chemical mutagenesis (Lellis et al., 2002; Pyott et al., 2016).

Because the introduction of the CRISPR-Cas9 cassette as a transgene might be controversial under certain regulations in some countries (**Table 2**), protocols have been developed to edit genomes without transgenes using guide RNA-Cas9 ribonucleoprotein complexes (Liang et al., 2017; Toda et al., 2019) or transient expression (Zhang et al., 2016).

**Table 2:** Regulation of genetically modified and genome edited plants across countries.

Country	Genetically modified plants^1^	Genome-edited plants^2^
Argentina	Regulated	Case-by-case, mostly non-regulated
Australia	Regulated	Non-regulated
Brazil	Regulated	Case-by-case, mostly non-regulated
Canada	Regulated	Regulated
Chile	Regulated	Case-by-case, mostly non-regulated
European Union	Regulated/opposed	Regulated/Opposed
India	Regulated	Regulated
Japan	Regulated	Non-regulated
Malaysia	Regulated	Regulated
Mexico	Regulated	Regulated
New Zealand	Regulated	Regulated
South Africa	Regulated	Regulated
Thailand	Regulated	Regulated
United States of America	Regulated	Non-regulated

^1^Refers to the final product containing transgenes, such as selection markers or other form of foreign DNA used during the process.

^2^Refers to the final product lacking transgenes that might have been used during the process.

Not all plant species are susceptible to *A. tumefaciens*. In species recalcitrant to Agrobacterium-mediated transformation, alternatives include *Rhizobium rhizogenes*-mediated or protoplast transformation. *R. rhizogenes* previously known as *Agrobacterium rhizogenes*, is a soil-borne gram-negative bacteria that causes hairy roots in plants. In most plant species, cells transformed with *R. rhizogenes* and its Ri plasmid differentiate into transformed roots, serving as a visual marker for marker-free screening and selection (Young et al., 2001; Bahramnejad et al., 2019). A comprehensive description of *R. rhizogenes* strains, binary vectors, and plants transformed using them is provided by (Bahramnejad et al., 2019). Examples of plants edited through CRISPR-Cas9 using *R. rhizogenes* include soybean (Du et al., 2016), tomato and rubber producing dandelion *Taraxacum kok-saghyz* (Iaffaldano et al., 2016).

## Protoplast or Zygote Transformation

For several plant species, including maize, soybean, wheat, rice, tomato, lettuce, arabidopsis, petunia, grapevine, apple, potato, and tobacco, protocols have been developed to isolate protoplast and transfect them with cassettes carrying CRISPR-Cas9 for genome editing purposes. Protoplast transfection has been used to rapidly optimize CRISPR-Ca9 parameters (Woo et al., 2015; Lin et al., 2018). However, isolation of single-protoplast has been used to regenerate stable transformants after transfection with cassettes carrying CRISPR-Cas9 or with ribonucleoprotein complexes assembled *in vitro* by synthesizing small guide RNAs and Cas9 protein. Gene editing using ribonucleoprotein complexes has the advantage of obtaining mutants without the presence of exogenous DNA. Preassembled Cas9-guide RNA ribonucleoproteins complexes can be delivered into protoplasts using polyethylene glycol-calcium-mediated transfection (Woo et al., 2015; Kim et al., 2017; Liang et al., 2017; Lin et al., 2018). To overcome the low efficiency of this approach, a protocol has been develop to transform plant zygotes by ribonucleoprotein complexes or by biolistic bombardment (Toda et al., 2019).

## *De Novo* Induction of Meristems

Delivering the CRISPR-Cas9 cassette into the germ line or protoplasts is technically challenging and inefficient. However, in dicotyledonous plants, those limitations might be eliminated through *de novo* induction of meristems. Developmental regulators and gene-editing components are delivered into somatic cells of whole plants. From treated tissue, shoots emerge that contain the targeted DNA modifications that are transmitted to the next generation (Maher et al., 2019).

## Applications of Genome Editing in Crop Improvement

Genome editing with CRISPR-Cas9 is amendable to edit any gene in any plant species. Because of its simplicity, efficiency, low cost, and the possibility to target multiple genes, it allows faster genetic modification than other techniques. It also can be used to genetically modify plants that were previously neglected. The potential that this represents for crop breeding and the development of sustainable agriculture is incommensurable (Cong et al., 2013; Mali et al., 2013; Zhang et al., 2017a; Toda et al., 2019; Wurtzel et al., 2019; Zhang et al., 2019b).

Impressive genetic modifications have been achieved with CRISPR-Cas9 to enhance metabolic pathways, tolerance to biotic (fungal, bacterial or viral pathogens), or abiotic stresses (cold, drought, salt), improve nutritional content, increase yield and grain quality, obtain haploid seeds, herbicide resistance, and others (**Table 1**). Notable cases include thermosensitive genic male sterility in maize (Li et al., 2017) and wheat (Okada et al., 2019), improved nutritional properties in sorghum and wheat (Li et al., 2018a; Zhang et al., 2018b), tolerance or resistance to pathogens (Zhang et al., 2017b; Pyott, 2016), and resistance to herbicides (Endo et al., 2016; Sun et al., 2016).

In potato CRISPR-Cas9 was used to knockout the gene encoding granule-bound starch synthase (GBSS) in one round of transfection resulting in the development of potato plants that produce amylopectin starch, a highly desirable commercial trait (Andersson et al., 2017). In cucumber CRISPR-Cas9 system was used to inactivate the eukaryotic translation initiation factor gene *elF*4E. The resulting non-transgenic homozygotic mutant plants were immune to Cucumber vein yellowing virus (Genus *Ipomovirus*) and resistant to the potyviruses Zucchini yellow mosaic virus and Papaya ring spot mosaic virus (Chandrasekaran et al., 2016). Engineering genetic resistance to viruses and other pathogens has immense potential to manage diseases for which no natural resistance has been detected, such as maize lethal necrosis disease and tomato brown rugose fruit virus (Luria et al., 2017; Garcia-Ruiz, 2018; Wamaitha et al., 2018).

## Human Exposure to CAS9 Proteins

In many bacteria and most archaea, CRISPR-Cas provides acquired immunity against viruses and plasmids by targeting nucleic acid in a sequence-specific manner (Horvath and Barrangou, 2010). Comparative genomic analyses revealed that CRISPR and genes coding for their associated proteins were present in diverse bacterial phylogenetic groups (Haft et al., 2005; Lillestol et al., 2006; Makarova et al., 2006). Since this adaptive immune system is useful for bacterial survival, it is likely to be present in all bacteria.

**Figure 3:**
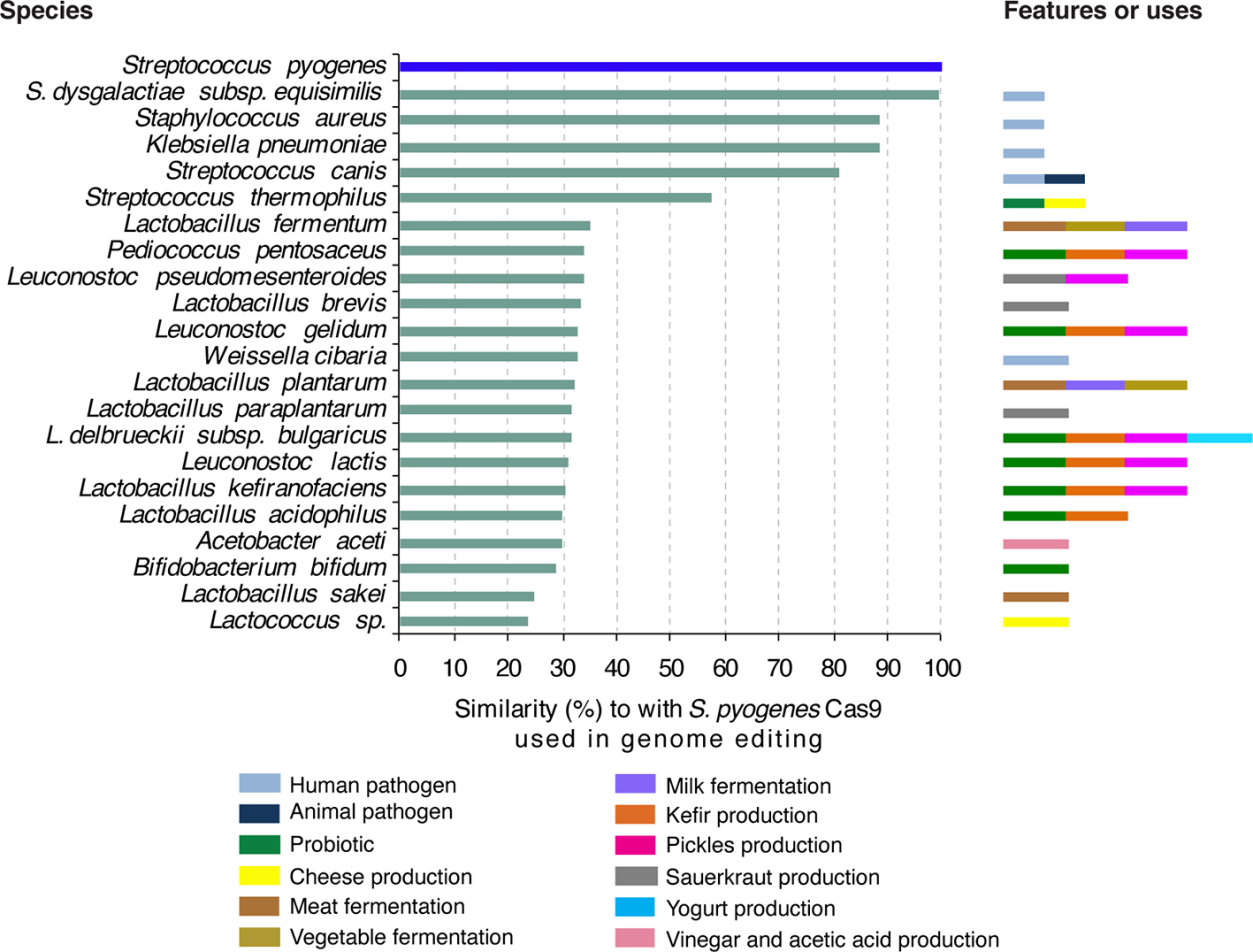
Bacteria frequently in contact with humans and similarity of their proteins to S. pyogenes Cas9 frequently used in plant genome editing. Amino acid sequence of *S. pyogene*s Cas9 was used to search for homologues proteins in GenBank. Proteins with more than 20% similarity are indicated. Features or anthropocentric use of each bacteria species are color-coded.

We compared the amino acid sequence of the Cas9 protein from *S. pyogenes* used in plant genome editing to proteins from bacteria to which humans are exposed through food consumption or in the environment. Results showed that Cas9 from *S. pyogenes* has 23% to 58% similarity to Cas9 protein from *Streptococcus thermophilus*, a bacterium widely used as a probiotic and in the production of cheese and yogurt (**Figure 3**). Additionally, Cas9 from *S. pyogenes* shares up to 35% similarity with Cas9 proteins from a wide range of bacteria used in food production such as *Lactobacillus plantarum* used to make cheese, yogurt, kefir and other fermented milk and meat products as well as fermented vegetables and beverages (Coloretti et al., 2007; Zago et al., 2011; Khemariya et al., 2016; Settachaimongkon et al., 2016; Sidira et al., 2017; Behera et al., 2018). *L. plantarum* is frequently encountered as a natural inhabitant of the human gastrointestinal tract, in which it is a transient guest acquirable through the diet (Vesa et al., 2000; De Vries et al., 2006). Additionally, *L. plantarum* is often used as a probiotic and can improve the balance of beneficial intestinal microflora (Nguyen et al., 2007; Nagpal et al., 2012; Kassayova et al., 2014).

Furthermore, Cas9 from *S. pyogenes* has homologues in diverse Gram-positive and Gram-negative bacteria that occupy very diverse niches throughout the human body (Louwen et al., 2014). Some are commensals and others are pathogenic bacteria. More than 80% amino acid sequence similarity was detected between Cas9 from *S. pyogenes* and that from human commensal and pathogenic bacteria such as *Streptococcus dysgalactiae subsp. equisimilis*, *Staphylococcus aureus*, *Klebsiella pneumonia* and *S. canis* (**Figure 3**).

These observations show that humans have been exposed to Cas9 proteins in their food and environment long before the development of genome editing. The biosafety risk of human exposure to the Cas9 used for plant genome editing needs further assessment (Pineda et al., 2019) and our results do not mean that potential human exposure to Cas9 used in genome editing is irrelevant.

## Biosafety Concerns About Genome-Edited Plants

Methodological, biosafety and social concerns remain about the use of genome editing in plants. They mostly are related to target gene site selection, guide RNA design, off-target effects, and the delivery method. The major concern is the risk of generating unwanted genetic changes in plants due to off-target mutations (Liang et al., 2018; Pineda et al., 2019). Fragments of the CRISPR-Cas9 might be degraded into filler DNA and inserted into expected and/or unexpected genomic positions during the DNA repair process (Gorbunova and Levy, 1997; Zhang et al., 2016). However, transgene integration and the risk of off-target mutations can be prevented by delivering *in vitro* pre-assembled CRISPR-Cas9 ribonucleoproteins (Malnoy et al., 2016; Svitashev et al., 2016; Zhang et al., 2016; Liang et al., 2018). This technique has already been used in several crop species but there are still some drawbacks in its application such as low stability, high costs and high levels of technical requirements, which need to be improved (Malnoy et al., 2016; Subburaj et al., 2016; Murovec et al., 2018).

Substantial work has also been done to minimize off-target effects of Cas9 itself, including improving RNA guide–design strategies, ribonucleoprotein delivery, protein engineering, using spatiotemporally controlled Cas9, and/or gRNAs through a plethora of chemical or environmental inducers, or using synthetic genetic circuits that modulate CRISPR function according to predefined logic (Svitashev et al., 2016; Liang et al., 2018). Base editing is also being modified to improve the specificity of base editors by limiting deaminase activity outside of Cas9 binding through the use of different deaminase effectors or rationally engineering the deaminase to decrease its DNA binding ability (Shimatani et al., 2017).

Other concerns about CRISPR-Cas9 technology are related to the Cas9 protein itself as it was shown to induce an immune response when delivered by adeno-associated virus in mice, making immunogenic side effects a concern (Chew et al., 2016). There are also concerns about the specificity of Cas9 and the limited number of sites which can be targeted due to the requirement of the PAM (Spencer and Zhang, 2017). Protein engineering efforts led to the identification of mutations in Cas9 that alter its PAM recognition and enhance its fidelity and recognize other motifs (Kleinstiver et al., 2015; Kleinstiver et al., 2016; Leenay and Beisel, 2017). Further modifications to Cas9 and guide RNA design, such as FokI fusions, paired nicking, and the use of truncated guide RNAs, have provided additional improvements to specificity (Wyvekens et al., 2015). Furthermore, Cas9 variants, Cas9 homologs derived from other bacteria, or novel Cas proteins such as Cpf1 nucleases can be used (Nakade et al., 2017; Pineda et al., 2019).

The societal concerns about genome editing stem in part from the lack of information about its principles and applications. A fundamental feature here is the distinction between genetically modified plants, transgenic plants, and genome edited plants (Garcia Ruiz et al., 2018; Eckerstorfer et al., 2019). Genome edited plants may or may not be transgenic. As indicated above, the transgene carrying the CRISPR-Cas9 cassette might be removed by gene segregation (**Figure 2**). If this is done, a genome-edited plant might be classified as non-transgenic. Educating the public on the principles of genome editing has the potential to correct and prevent the spread of misconceptions (Garcia Ruiz et al., 2018; Eckerstorfer et al., 2019).

## Regulation of Genome-Edited Crops

The term genetically modified refers to plants whose genome has been modified in a way that would not have been occurred naturally (Wang et al., 2016b; Duensing et al., 2018; Friedrichs et al., 2019).

In contrast, gene editing refers to DNA modifications similar to those potentially generated naturally (deletions, nt substitutions, insertions) of by conventional plant breeding (Nature Plants Editorial, 2018). The basis to regulate the release and international trade of living genetically modified organisms were established in the Cartagena Protocol on Biosafety. However, production, consumption, and regulation of genetically modified plants have followed contrasting patterns. While some countries reject consumption and ban production, others openly grow and consume them (Garcia Ruiz et al., 2018).

Regulation of genome-edited plants follows two frameworks. Some countries regulate the process, while others regulate characteristics of the final product (Eckerstorfer et al., 2019; Van Vu et al., 2019). While some countries have established biosafety regulations for genome edited plants, or declared their deregulation (**Table 2**), most countries have not yet established their position (Eckerstorfer et al., 2019). Challenges in regulating plant genome editing include market access, and addressing the societal concerns about its biological safety without limiting the development of the technology (Kupferschmidt, 2018; Eckerstorfer et al., 2019). Transgene-free, genome-edited plants are similar to varieties containing genetic variations created naturally (**Figure 2**). Therefore, commercialization of genome edited plants or their products might bypass the strict biosafety regulations required for transgenic plants (Tuteja et al., 2012; Van Vu et al., 2019).

The United States Department of Agriculture (USDA) declared in March 2018 that genome editing is the equivalent of conventional breeding in some instances and therefore does not require regulatory oversight within the American regulatory framework (Waltz, 2016a). A mushroom engineered to resist browning and a waxy corn engineered to contain starch composed exclusively of amylopectin are the first CRISPR edited crops to be approved for commercialization in the USA with no regulations (Waltz, 2016b). The decision not to regulate was based on the fact that no foreign DNA (transgene) was inserted during editing and that the resulting change did not involve resistance to pesticides or herbicides.

Canada, on the other hand, has remained committed to the scientific principles laid down in its domestic regulatory framework for plants with novel traits established 25 years ago. Canada's policy states that any gene editing technology that creates a novel product is subject to additional regulatory oversight on allergenicity, toxicity and impacts on non-target organisms (Smyth, 2017). Two products obtained by gene editing have been approved in Canada, non-browning apples and non-dark spots potatoes (Waltz, 2016b). The approval was granted after a lengthy evaluation process that determined that the changes made to the apples and the potatoes did not pose a greater risk to human health than apples and potatoes currently available on the Canadian market (Waltz, 2016b).

Argentina has developed a functional regulatory system for the approval of genome-edited products (Whelan and Lema, 2015). The regulatory system was developed to be consistent with the Cartagena Protocol on Biosafety and relies on case-by-case assessment. If a transgene technology was used in the development of a product, where the final product is free of the transgene, then this product can be classified as nontransgenic. Chile and Brazil followed Argentina's lead. Chile signed a normative resolution in 2017 while Brazil published a resolution in January 2018 (Duensing et al., 2018). Both regulate gene-edited products on a case-by-case basis and exempt them from regulation when there is no insertion of transgenes.

Meanwhile, European Union (EU) countries remain politically opposed to genetically modified crops (Waltz, 2016b). On July 2018, the Court of Justice of the European Union (ECJ) ruled that gene-edited crops should be subject to the same stringent regulations as conventional genetically modified (GM) organisms. In its ruling, the ECJ determined that only mutagenesis techniques that have conventionally been used in a number of applications and have a long safety record are exempt from this rule.

In Australia, the Gene Technology Act (GT Act), introduced in 2000, stipulates that a GMO is an organism produced by any technique that modifies genes or other genetic material. In 2001, the Gene Technology Regulations were introduced. Schedule 1 of these regulations, specifies that organisms resulting from an exchange of DNA in which the donor species is also the hosts species and the vector DNA does not contain heterogenous DNA as not GMOs. In October 2019, an amendment to schedule 1 came in effect. The amendment excludes organisms modified through CRISPR-Cas9 and other unguided repair of site-directed nuclease activity (SDN), from being regulated as GMOs. The amendment also indicates that organisms generated in the intermediated steps of the SDN method are deemed non GMOs if 1) no nucleic acid template is supplied to guide genome repair through homology-directed recombination, and 2) the organism has no other modifications as a result of the gene technology (Eckerstorfer et al., 2019).

In New Zealand, importation, development, field testing, and release of GMOs genetically modified are regulated by the Hazardous Substances and New Organisms Act 1996 (HSNO Act). The country has the most rigorous and comprehensive process for regulation of GMOs. As a result of that, no GMO commercial crops are grown in the country and no GM meat or fresh produce is sold in the country. Furthermore, processed food that contains imported GM ingredients is tested for safety and should be labeled as so. In 2016, the HSNO Act was amended with an article stating that plant breeding by genome editing is subject to the same regulations as the GMOs (Shimatani et al., 2017).

India's regulatory process for research, development and use of GMOs and their products, including new gene technologies was established in 1989. The Food Safety and Standards Authority of India define genetically engineered or modified food as “any food or food ingredient composed or containing genetically modified or engineered organisms obtained through modern biotechnology, or food and food ingredients produced from but not containing genetically modified or engineered organisms obtained through modern biotechnology”. Thus all new technologies including CRISPR-Cas9 gene technologies (including genome editing) are still regulated within the existing regulatory framework (Friedrichs et al., 2019).

Japan's Ministry of Health, Labor and Welfare (MHLW) has recently declared that foods derived from genome editing technologies which do not contain transgenic genes and/or fragments of transgenic genes are not considered GMOs and are not subject to regulations as long as the DNA double-strand break induced by the genetic engineering method is either a base-pair deletion, a naturally occurring gene deletion and/or a concomitant insertion of one to several base pairs. The new MHLW's policy also indicates that off-target mutations in GE foods should not be of concern as they can also be observed in multiple locations in the genome of crops produced by traditional breeding (South et al., 2019; Van Vu et al., 2019).

It seems that the decision to regulate or not regulate GE crops and foods depends mainly on the type of GMO regulatory system already in place in the country. Countries that have adopted a process-based GMO regulatory system and consider that products made using the regulated process are fundamentally different or more risky than similar products made using other methods will likely regulate GE crops and foods under the GMO laws. On the other hand, countries who follow a product-based regulatory system and regulate based on the characteristics of the final product rather than the process by which it was made might not regulate GE crops and foods under GMO laws. Countries, such as Malaysia and Thailand, who adopt a dual product and process approach will also likely to regulate GE crops and foods under GMO laws (Friedrichs et al., 2019).

## Future Perspectives

Due to the many practical applications related to food production, genome editing can and will be used to solve agricultural issues that directly affect food security, such a citrus greening disease (Taylor et al., 2019), and the high yield losses in C3 plants, such as rice and barely, due to inefficient photorespiration in these crops. A recent report described the construction of three synthetic glycolate metabolic pathways in tobacco chloroplasts with the aim of improving the plant's photosynthetic efficiency. Flux through the synthetic pathways was maximized by inhibiting glycolate export from the chloroplast using RNA interference to down-regulate a native chloroplast glycolate transporter. In the transgenic tobacco plants, the photosynthetic yield increased by 20% while biomass productivity improved by more than 40% (South et al., 2019). While this study did not use genome editing technology and was carried out in *Nicotiana tabacum*, a model plant, the concept can be applied easily and successfully in staple crops using CRISPR-Cas9. Successful improvement in photorespiration efficiency in crops such as maize, rice and wheat, has the potential to substantially increase food production for the growing worldwide population while using the same cultivation areas and without having to destroy more forest areas for agricultural purposes. Producing crops with better quality food through genome editing will also help achieve food security (Li et al., 2018b; Narayanan et al., 2019).

## Conclusions

Genome editing in general, and CRISPR-Cas9 in particular, is a revolutionary tool that can impact science, food production, and society. CRISPR-Cas9 has great potential for transforming agriculture by making plants tolerant to biotic and abiotic stresses and improving their nutritional value and yield. These attributes are necessary to meet the demand of an increasing world population. In order to be able to effectively and durably use this technology in crop improvement, the scientific community needs to address the various biosafety and societal concerns about it. There is also a need to re-evaluate the regulations of genome-edited plants and to educate the general public about their properties.

## Author Contributions

HG-R conceived the study. KE-M, MM-F, and HG-R performed the analysis. KE-M, MM-F, and HG-R wrote the paper.

## Funding

This research was supported by NIH grant R01GM120108 to HG-R and by the Nebraska Agricultural Experiment Station with funding from the Hatch Act (Accession Number 1007272) through the USDA National Institute of Food and Agriculture. Open access costs were provided by the same grant. MM-F was the recipient of a graduate student fellowship from CONACYT, Mexico.

## Conflict of Interest

The authors declare that the research was conducted in the absence of any commercial or financial relationships that could be construed as a potential conflict of interest.
